# Experimental and Simulated Investigations of Thin Polymer Substrates with an Indium Tin Oxide Coating under Fatigue Bending Loadings

**DOI:** 10.3390/ma9090720

**Published:** 2016-08-24

**Authors:** Jiong-Shiun Hsu, Chang-Chun Lee, Bor-Jiunn Wen, Pei-Chen Huang, Chi-Kai Xie

**Affiliations:** 1 Department of Power Mechanical Engineering, National Formosa University, Yunlin 632, Taiwan; jshsu@nfu.edu.tw (J.-S.H.); sq1356@livemail.tw (C.-K.X.); 2 Department of Mechanical Engineering, National Chung Hsing University, Taichun 402, Taiwan; mars420225@gmail.com; 3 Department of Mechanical and Mechatronic Engineering, National Taiwan Ocean University, Keelung 202, Taiwan; jiunner@gmail.com

**Keywords:** ITO/PET film, fatigue bending test, sheet resistance, optical transmittance, energy released rate

## Abstract

Stress-induced failure is a critical concern that influences the mechanical reliability of an indium tin oxide (ITO) film deposited on a transparently flexible polyethylene terephthalate (PET) substrate. In this study, a cycling bending mechanism was proposed and used to experimentally investigate the influences of compressive and tensile stresses on the mechanical stability of an ITO film deposited on PET substrates. The sheet resistance of the ITO film, optical transmittance of the ITO-coated PET substrates, and failure scheme within the ITO film were measured to evaluate the mechanical stability of the concerned thin films. The results indicated that compressive and tensile stresses generated distinct failure schemes within an ITO film and both led to increased sheet resistance and optical transmittance. In addition, tensile stress increased the sheet resistance of an ITO film more easily than compressive stress did. However, the influences of both compressive and tensile stress on increased optical transmittance were demonstrated to be highly similar. Increasing the thickness of a PET substrate resulted in increased sheet resistance and optical transmittance regardless of the presence of compressive or tensile stress. Moreover, J-Integral, a method based on strain energy, was used to estimate the interfacial adhesion strength of the ITO-PET film through the simulation approach enabled by a finite element analysis.

## 1. Introduction

Indium tin oxide (ITO) film is transparent and has a low electrical resistance. Recently, ITO film has been increasingly deposited on transparent flexible polyethylene terephthalate (PET) substrates as the electrode for a wide range of items such as displays, sensors, and solar cells [1,2,3,4]. Compared with a PET substrate exhibiting a lower Young’s modulus, the brittle ITO film deposited on PET has a large Young’s modulus [5]. From a mechanical perspective, severe residual stress is generated within an ITO film because of the substantial elastic mismatch between the PET substrate and ITO film [6,7,8]. Consequently, failure may possibly be generated within the ITO film, thereby degrading the electrical property and the optical transmittance of the ITO film.

Because the stress-induced failure problem plays a pertinent role in the operational capacity of relevant products, many researchers have investigated the foregoing problem. Grego et al. [9] developed an experimental technique called the “x-y-θ” geometry to perform bending testing on an ITO-coated PET specimen and discussed the relationship between the bending radius and electrical resistance of the ITO film. Lin et al. [10] studied the deflection effect on the mechanical and optoelectronic properties of the ITO film deposited on a PET substrate by using pulsed magnetron sputtering. Lan et al. [11] used thermionic emission to improve the durability of an ITO film deposited on a PET substrate under mechanical bending, causing its optical and electrical properties to simultaneously increase. Hamasha et al. investigated the stability of an ITO film deposited on a PET substrate, subjecting it to strength, bending fatigue, thermal aging, and cycling [12,13,14]. Lee and Liu [15,16] developed theoretical solutions for flexible electronics under torsion loading.

Generally, the materials subjected to compressive and tensile stresses have various mechanical responses [9,10,11]. For this reason, this study explored the influences of compressive and tensile stresses on the mechanical stability of an ITO film deposited on a PET substrate. A specially designed mechanism integrated with two servo motors was established to perform the cycling bending testing on the ITO-coated PET substrate. In our designed mechanism, the stress state of the ITO film could be altered from compressive to tensile stress by changing the placement orientation of the ITO-coated PET substrate. An optical microscope was used to observe the failure scheme of the ITO film, which resulted from the cycling bending load. A four-point probe was employed to measure the sheet resistance of the ITO film to clarify the influences of compressive and tensile stresses on the sheet resistance and failure configuration of the ITO film. Finally, a spectrometer was applied to measure the transmittance of the ITO-coated PET substrate to understand the relationship between previous discoveries and optical transmittance.

## 2. Experimental Details of the ITO-PET Film

### 2.1. Establishment of the Bending Test Mechanism and Bending Experiment

**Figure 1:**
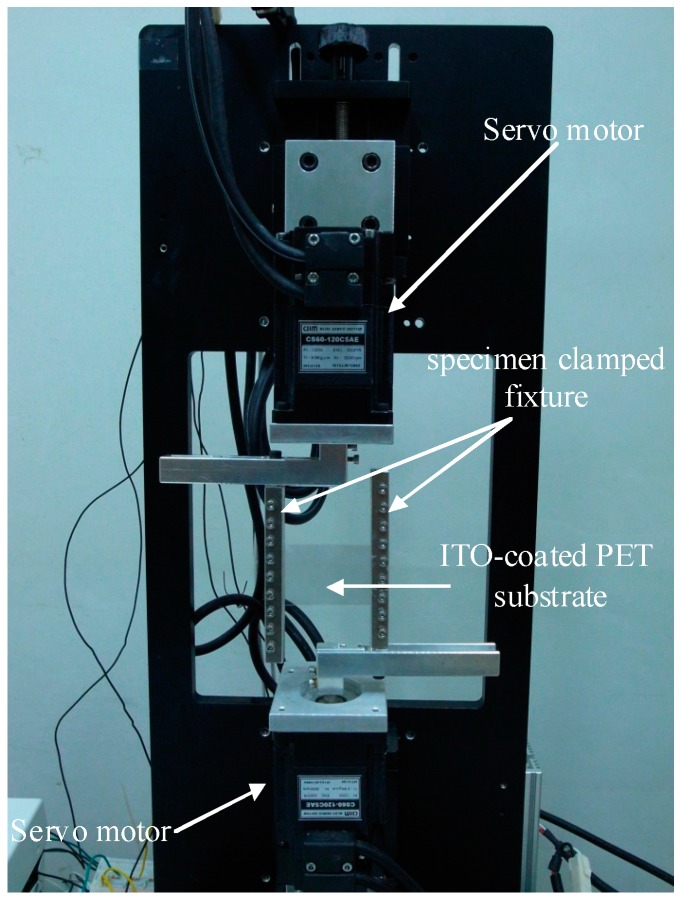
Image of the testing mechanism used in the ITO-PET bending experiment.

**Figure 2:**
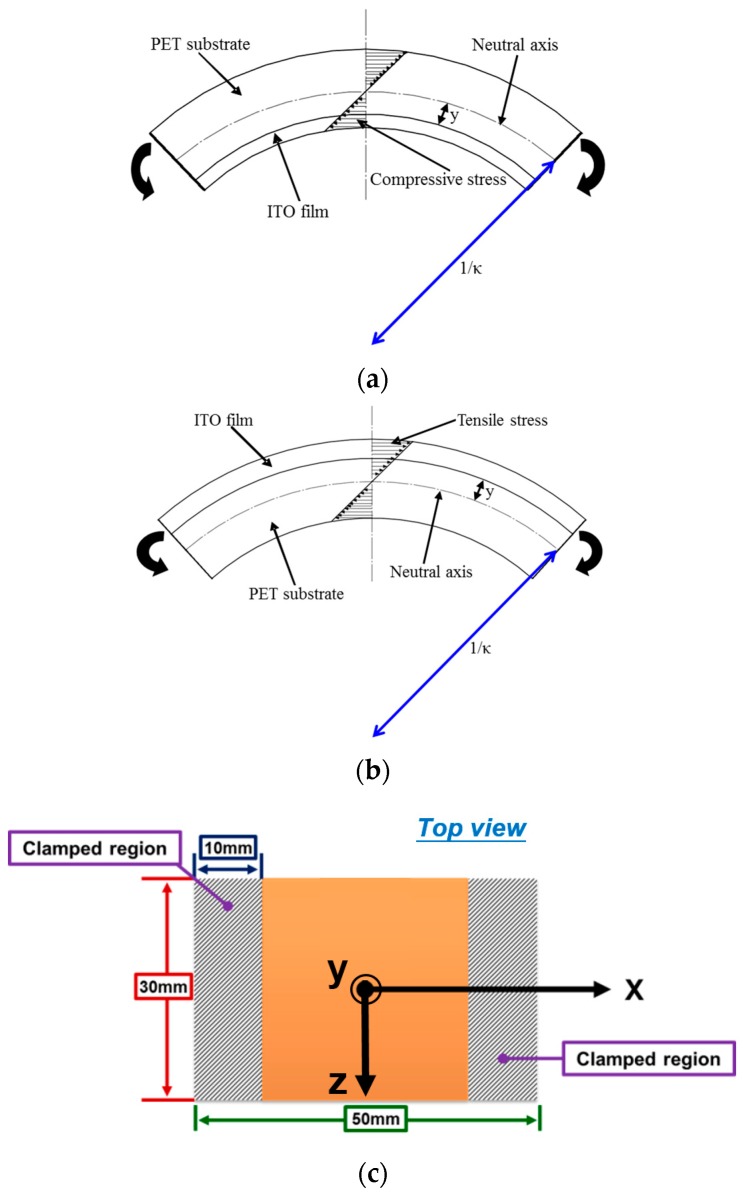
Stress states of the ITO-coated PET substrate and the geometry of testing specimens: (**a**) compressive stress; (**b**) tensile stress; (**c**) the geometry of the test specimens.

In this study, a self-designed bending testing mechanism was proposed and demonstrated. The image of the bending testing mechanism is shown in Figure 1, in which two servo motors (CSBL900, CSIM Inc., Taipei, Taiwan) were placed against the upper and bottom portions of the mechanism, respectively. A computer numerical control was used to manufacture a specimen-clamped fixture, as indicated in Figure 1. LABVIEW (National Instruments, Austin, TX, USA) was used to control the rotated degree of the servo motors. When these two motors were rotated, they could apply a bending force on the clamped ITO-coated PET substrate, as illustrated in Figure 1. Hence, the cycling bending test could be performed on an ITO-coated PET substrate by using this mechanism. Additionally, the stress state of the ITO film could be altered from compressive stress to tensile stress by changing the placement orientation of the ITO-coated PET specimen, as depicted in Figure 2a,b.

For the test specimens, 22-nm-thick ITO films were deposited on two PET substrates with two different thicknesses of 125 and 188 μm by using pulsed DC sputtering. The detailed process conditions and post annealed treatments were the same as those described in [17]. The geometry of the testing specimen is illustrated in Figure 2c; the shaded area represents the clamped region. To avoid the residual stress influence of ITO-coated PET substrates, all substrates were heated by a heater at 85 °C for 1.5 h to release the residual stress. The testing specimen was then fixed to start the cycling bending testing, as shown in Figure 1. The rotated degrees of the two servo motors were both fixed at 72° and the bending radius of the curvature under the bending test was 39.8 mm. The bending cycle ranged from 2000 to 12,000 times to observe the fatigue property of the studied ITO-PET film.

### 2.2. Sheet Resistance Measurement

A four-point probe (MCP-T370, Mitsubishi Chemical Analytech Co., Tokyo, Japan) was employed to measure the sheet resistance of the ITO film under the fatigue bending test. Because the compressive or tensile stresses of the ITO film were achieved through application of the bending loading, a four-point probe was used to perform measurements along the longitudinal direction (i.e., x-direction, transverse direction, and z-direction), as depicted in Figure 2c, of the sheet resistance to explore the bending effects on the longitudinal and transverse directions of the ITO film.

### 2.3. Observation of ITO Film Failure and Optical Transmittance Measurement

An optical microscope (M835, Microtech Instruments Inc., Eugene, OR, USA) was applied to observe the failure mechanism of the ITO film. Because the specimens experienced cycling of compressive or tensile stress, the specimens were unavoidably subjected to warpage from the residual stress. To effectively observe the failure of the ITO film, an ITO-coated PET substrate was placed between two carry sheet glasses to flatten it, thereby enabling the capture of the failure image. Moreover, a spectrometer (USB 4000, Ocean Optics, Dunedin, FL, USA) was used to measure the optical transmittance.

## 3. Experimental Results and Discussion

**Figure 3:**
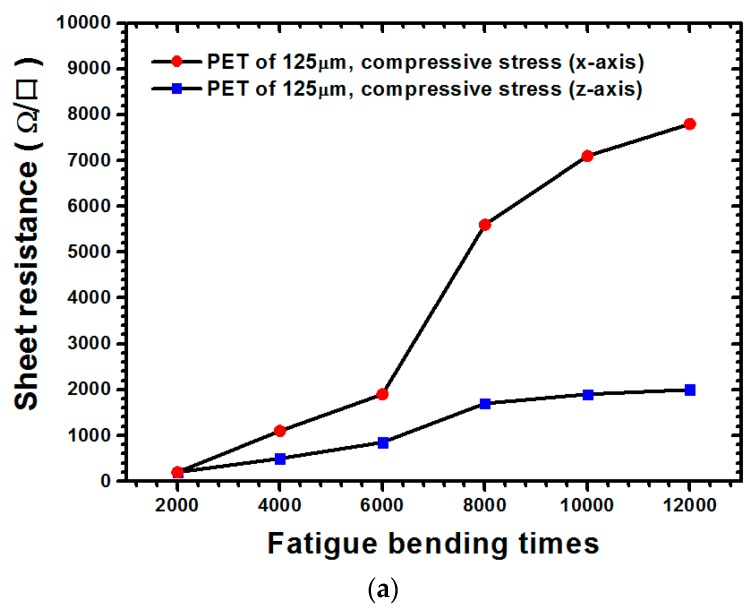

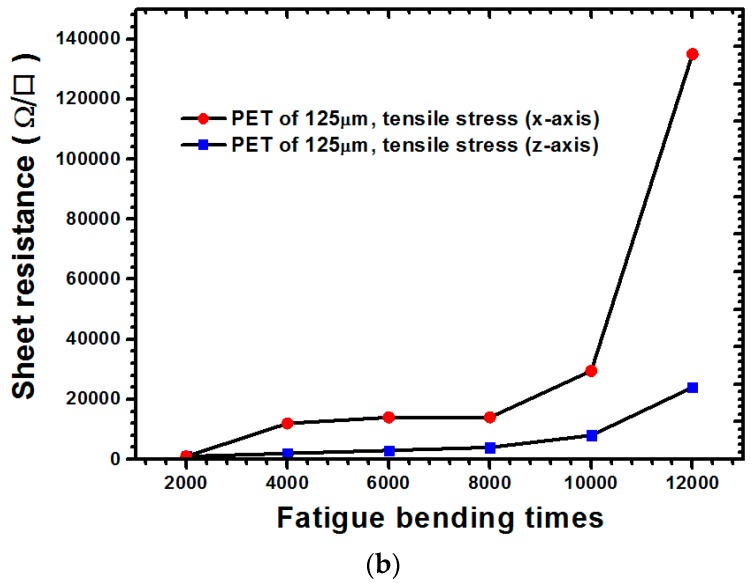
Electrical measurement results for the ITO-PET film with a 125-μm substrate thickness under various load types: (**a**) compressive stress; (**b**) tensile stress.

**Figure 4:**
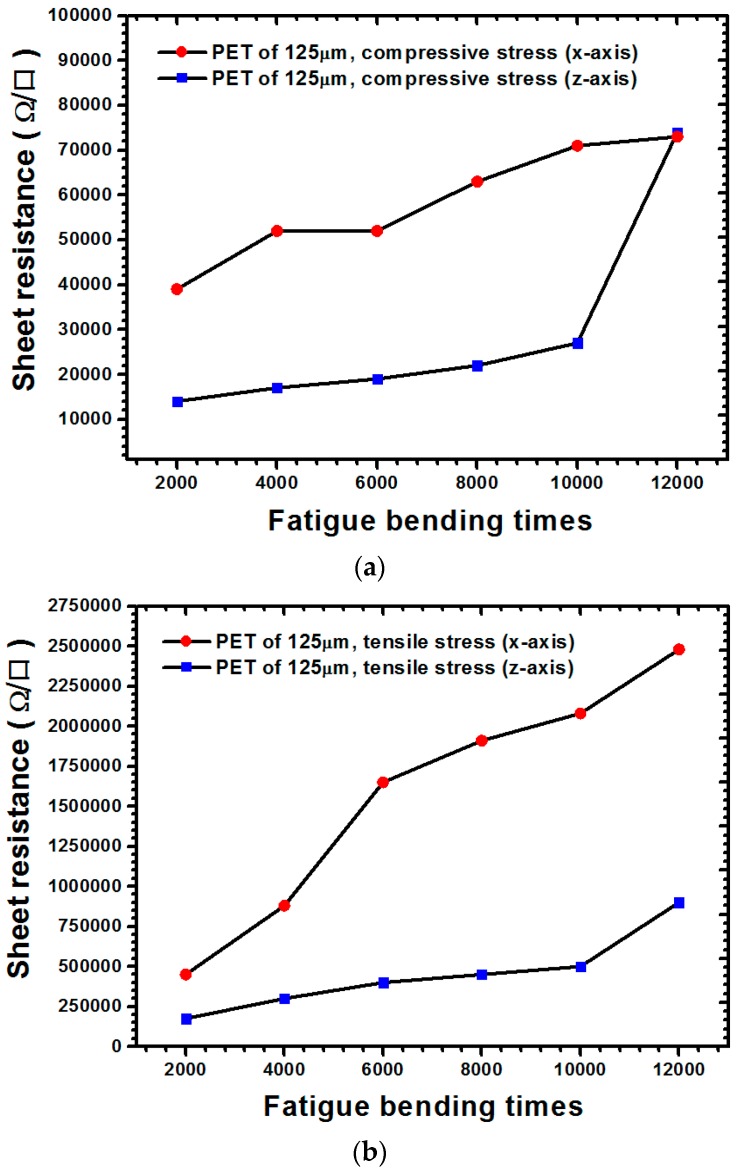
Electrical measurement results for the ITO-PET film with a 125-μm substrate thickness under various load types: (**a**) compressive stress; (**b**) tensile stress.

The measurement results of the sheet resistance of the ITO film with 125-μm thickness under various types of stresses are illustrated in Figure 3. For cases where the ITO film was subjected to compressive stress (Figure 3a) when the 12,000-times bending cycle was applied, the sheet resistance values along the x-direction and their increasing rate were both larger than those of the z-direction. When the ITO film underwent tensile stress (Figure 3b), the sheet resistance along the x-direction dramatically increased and the bending cycle increased to 12,000 times. As illustrated through a comparison of Figure 3a,b, regardless of the x- or z-direction, the sheet resistance values of the ITO film subjected to tensile stress were at least one order larger than those of the film subjected to compressive stress, thereby revealing that tensile stress can easily increase sheet resistance compared with compressive stress. The measurement results for the 188 μm thickness are shown in Figure 4. Unlike the previous result obtained for a PET substrate with a thickness of 125 μm, when the bending cycle was increased to 12,000 times, a small increase in sheet resistance was observed along the x-direction. However, the increase along the z-direction was significantly greater than that along the x-direction (Figure 4a). Regarding the state of tensile stress for the PET substrate with a 188-μm thickness (Figure 4b), the sheet resistance values along the x-direction were all larger than those along the z-direction, with the bending cycle increasing up to 12,000 times. Figure 4a,b indicate that because the sheet resistance values under tensile stress were larger than those under compressive stress, tensile stress led to a sheet resistance increase of the ITO film more easily than did compressive stress. Furthermore, as demonstrated through a comparison of Figure 3 and Figure 4, increasing the thickness of the PET substrate evidently increased the sheet resistance of the ITO film.

**Figure 5:**
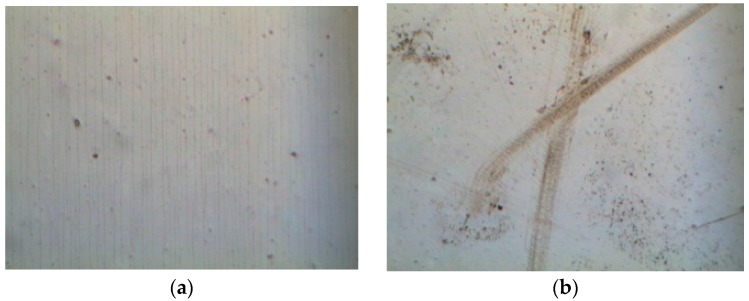
Optical images of ITO films deposited on PET substrates with a 125-μm thickness and 12,000-times bending cycle subjected to (**a**) compressive stress and (**b**) tensile stress.

**Figure 6:**
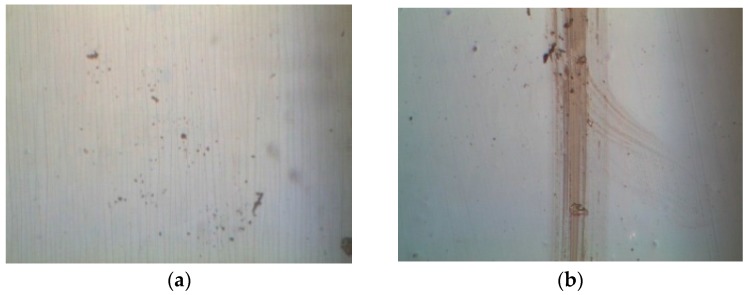
Optical images of ITO films deposited on PET substrates with a 188-μm thickness and 12,000-times bending cycle subjected to (**a**) compressive stress and (**b**) tensile stress.

**Figure 7:**
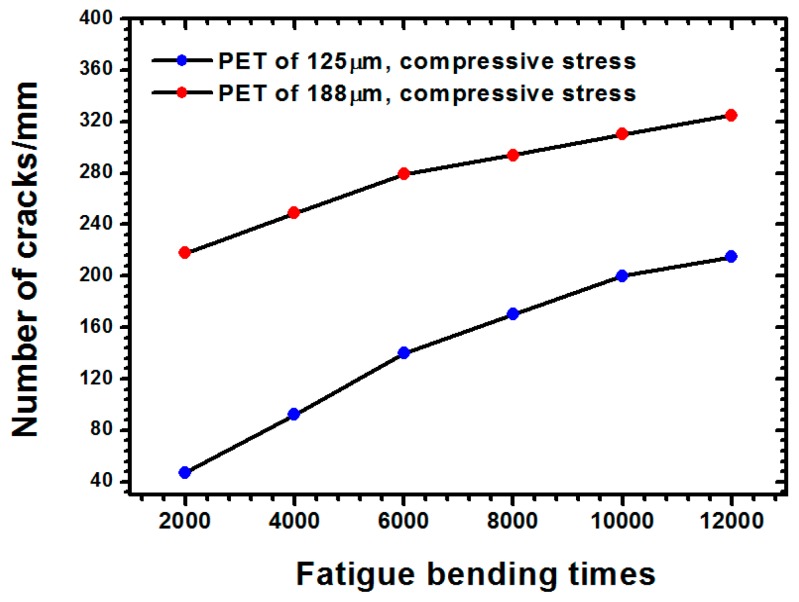
Comparison of the number of microcracks per millimeter for different PET substrates under compressive stress.

The optical images of the PET substrate with a 125-μm thickness subjected to compressive stress are depicted in Figure 5a, in which parallel microcracks lie along the y-direction (i.e., transverse direction). Additionally, the number of microcracks increased with an increasing bending cycle and when the bending cycle was fixed at 12,000 times under compressive stress. Figure 5b indicates the optical images of the PET substrate (125-μm thickness) were subjected to tensile stress. However, the microcracks that appeared in the compressive stress state are unobservable in Figure 5b. When the bending cycle was increased to 12,000 times, two intersecting grooves (Figure 5b) caused a dramatic increase in the sheet resistance of the ITO film, as indicated in Figure 3b. The optical images of the ITO film deposited on a PET substrate (188-μm thickness) subjected to compressive and tensile stresses are shown in Figure 6a,b, respectively. In Figure 6a, many parallel microcracks lie along the z-direction that were generated even at a 12,000-times bending cycle. We measured the number of microcracks per millimeter in Figure 5a and Figure 6a to quantitatively evaluate the influence of PET thickness on the number of the formed microcracks. Figure 7 shows the comparison of the number of microcracks per millimeter for different PET substrates under compressive stress. Figure 7 depicts that the number of microcracks per millimeter increased with an increasing bending cycle, whether the PET thickness was 125 μm or 188 μm. In addition, the number of microcracks for the PET substrate with a 188-μm thickness was higher than that of the PET substrate with a 125-μm thickness. The aforementioned results can explain the phenomenon mentioned at the end of the preceding paragraph; increasing the thickness of the PET substrate more notably increased the sheet resistance under the compressive stress state.

**Figure 8:**
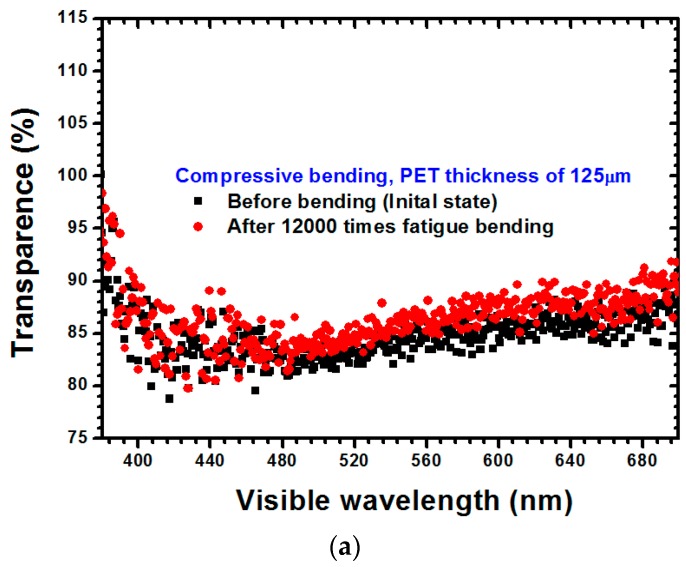

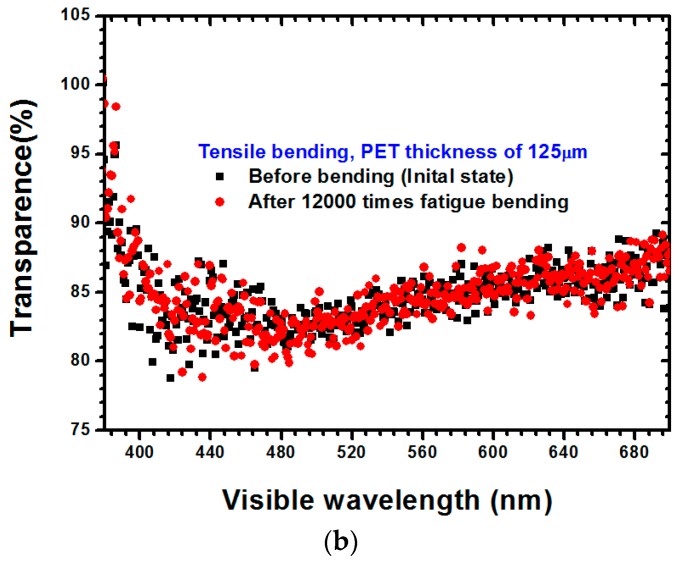
Optical transmittance measurement results for the ITO-PET film with a 125-μm substrate thickness under (**a**) compressive stress and (**b**) tensile stress.

**Figure 9:**
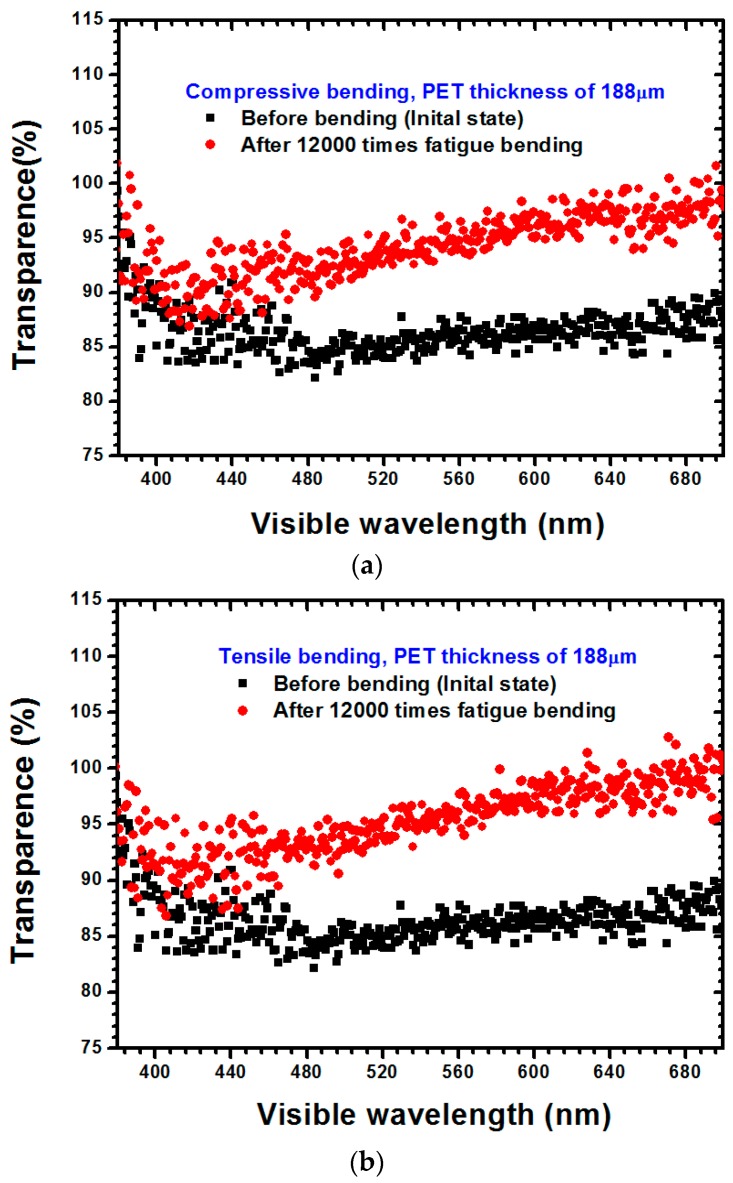
Optical transmittance measurement results for the ITO-PET film with a 188-μm substrate thickness under (**a**) compressive stress and (**b**) tensile stress.

The optical transmittances of PET substrates with a 125-μm thickness subjected to compressive and tensile stresses are depicted in Figure 8a,b, respectively. The optical transmittance of the ITO film was smaller than that of the PET substrate, thus facilitating light transference through the ITO-coated PET substrate when the failure occurred within the ITO film, indicating that the optical transmittance increased along with the increase of the failure degree within the ITO film. Similar results can be also observed in Figure 9a,b for the PET substrate with a thickness of 188 μm. The reason for these results is identical to that of the results for the PET substrate with a 125-μm thickness. As observable in Figure 8 and Figure 9, the difference between the influences of compressive stress and tensile stress on the optical transmittance of the ITO films was not obvious. Additionally, regardless of if it was under compressive stress or tensile stress, increasing the thickness of a PET substrate caused the optical transmittance to increase. According to Gere and Timoshenko [18] and the results revealed in Figure 2, the uniaxial stress can be obtained as follows:

(1) $$\sigma_{x}=E\kappa$$

where σ_x_ is the uniaxial bending stress along the x-axis direction, *E* is the Young’s modulus of the concerned thin film, κ is the bending curvature, and *y* is the distance from the neutral axis. From Equation (1), the uniaxial stress based on a thicker testing sample is determined to be higher than the others. Therefore, the electrical and optical transmittance performances as well as the number of microcracks of the testing sample increased at both bending compressive and tensile stress states as the thickness of the PET substrate became larger.

## 4. Validation and Simulation Approaches of Fracture-Based Analysis for ITO-PET Thin Films

**Figure 10:**
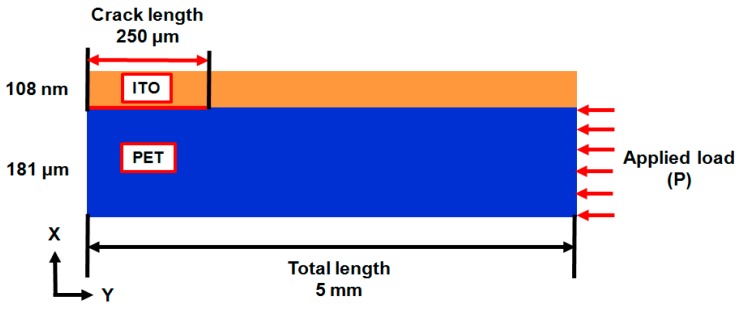
Illustration of ITO-coated PET substrate with an interfacial crack.

The brittle thin film fracture deposited on a compliant substrate is a major concern and a reliability problem for a stacked multilayer thin film structure. In addition, the interfacial adhesion strength between the coated film and substrate also plays a crucial role in mechanical reliability. According to the foregoing explanation, the fracture toughness induced by buckling delamination under a compressive load must be analyzed. To validate that the energy release rate obtained by the proposed fracture-based approach was satisfactory and reliable, the interfacial strength of the ITO-PET film, which was considered as the interfacial crack measured by Chen et al. [19], was selected for validation. The dimensions and materials used in the energy release rate estimation of the selected ITO-PET film are illustrated in Figure 10. The proposed ITO-PET film had the following characteristics: 108-nm- and 181-μm-thick ITO and PET substrates, and crack-total lengths of 250 μm and 5 mm, respectively. The uniform load (P) was applied along the y-direction of the PET to induce the buckling of the ITO film coated with the substrate. Moreover, the properties of the material used in the interfacial strength estimation of the ITO-PET film are listed in Table 1.

### 4.1. Energy-Based Method of Adhesion Strength Estimation: J-Integral Approach

In general, adhesion strength is difficult to estimate using specific stress components because of the mixed mode of fracture force that is induced. Therefore, an energy-based approach method J-Integral was assumed to estimate the critical strain energy release rate (*G_c_*). The standard formula of J-Integral is provided by [20,21] as follows:

(2) $$J=\int_{\Gamma }(Wdy-T\frac{\partial u}{\partial x}ds)$$

where *W* denotes the strain energy density per unit capacity and *T* and *u* are the surface traction and displacement vectors alongside the *Γ* curve, respectively. Furthermore, *Γ* is an arbitrary contour path around the crack tip and *ds* is an infinitesimal section of the contour length along *Γ*. Because the J-Integral is a path-dependent approach for a finite element analysis (FEA) simulation and a decent selection of the integral path is required for the calculation of interfacial adhesion among stacked thin films in a FEA model, a stable J-Integral path definition in the FEA when the ratio of Track2 was divided by Track1 approached infinity [21].

### 4.2. Validation of Interfacial Adhesion Strength of Selected ITO-PET Film

**Figure 11:**
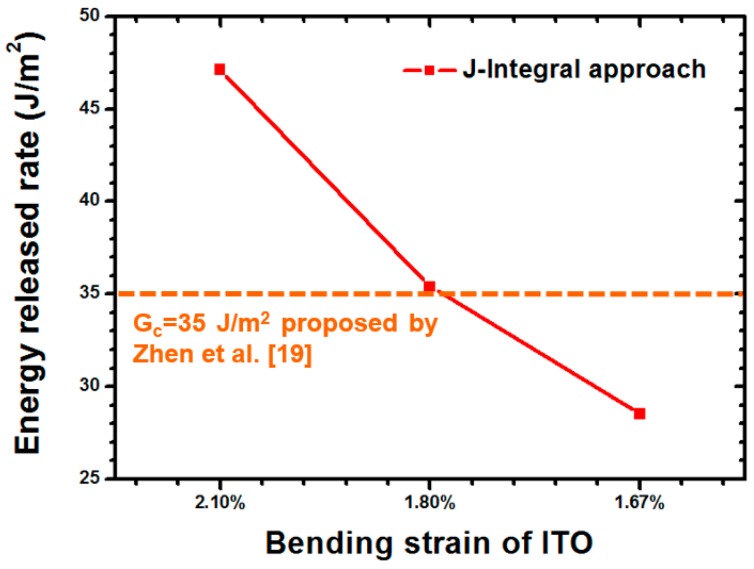
Energy release rate estimated using the J-integral approach in which various ITO bending strains were applied.

The delamination toughness of the ITO-PET film under a compressive load was estimated at approximately 35 J/m^2^ [19]. The testing results of the J-Integral approach are shown in Figure 11. Moreover, the bending strain of the ITO was obtained to ensure the accuracy of the simulation approach. The results revealed that the critical energy release rate (G_c_) of the ITO-PET interface and bending strain of the ITO were 35.4 J/m^2^ and 1.8%, respectively. The extracted G_c_ and bending strain of the ITO demonstrated good agreement with the results reported by Zhen (G_c_ = 35 J/m^2^, bending strain of ITO = 1.7%). Thus, a reliable simulation approach was demonstrated to estimate the adhesion strength.

### 4.3. Influence of Crack Length, PET Thickness, and ITO Thickness on Energy Release Rate Estimation

#### 4.3.1. Influence of Crack Length on J-Value Predication

**Figure 12:**
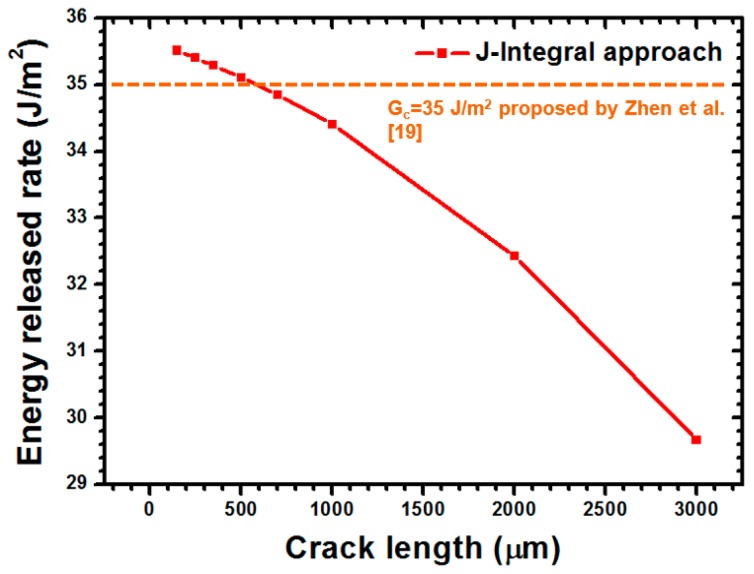
Curve of crack length versus G-value for different interfacial crack lengths in the FEA method.

For the predication of interfacial adhesion among the analyzed ITO-PET films, the strain energy release rate (G) of the concerned interface was estimated using the J-Integral approach. As depicted in Figure 12, the crack length dependence G-value was predicated when the various crack lengths of 150, 250, 350, 500, 700, 1000, 2000, and 3000 μm were applied. A significant reduction in the G-value was observed when the crack length was larger than 700 μm. This phenomenon can be explained as follows. The uniform load applied on the side of the PET substrate had difficulty inducing the buckling delamination because of the extended crack length. When the crack length ranged from 150 μm to a milli-level of 3000 μm, a reduction in the estimated G-value from 35.51 J/m^2^ to 29.67 J/m^2^ was achieved.

#### 4.3.2. Thickness Effect of PET Compliant Substrate

**Figure 13:**
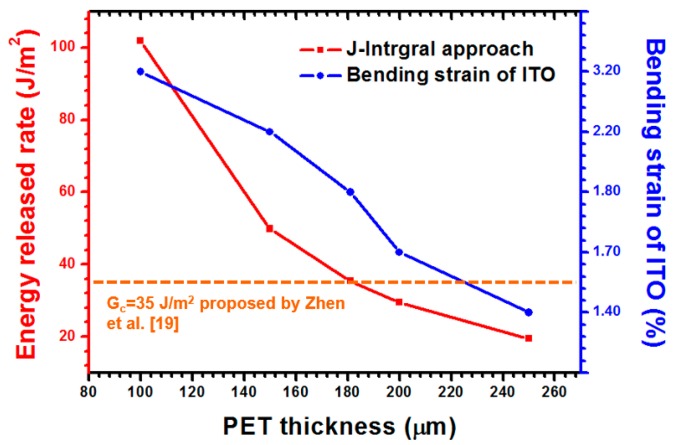
Comparison of energy release rates of the ITO–ET interface and ITO bending strain observed on an ITO surface with consideration of various PET thicknesses.

The thickness effect of the PET is shown in Figure 13. Several ITO thicknesses were analyzed, comprising 100, 150, 181, 200, and 250 μm. A thicker PET induces a lower strain and energy release rate than do PETs with other thicknesses and identical interfacial crack lengths. A significant reduction of the interfacial cracking energy of the ITO-PET films was observed when the PET thickness ranged from 100 μm to 250 μm. Hence, a reduction of the energy release rate from 101.91 J/m^2^ to 19.42 J/m^2^ was predicted for the PET substrate within said range of thicknesses. A reduced bending strain of the ITO that ranged from 3.2% to 1.4% was observed. The increased PET thickness induced bending strain in the ITO. This behavior could be attributed to the higher structural stiffness and higher distance from the neutral plane to the surface of the ITO as a result of the increased thickness. Thus, the thick PET substrate is suggested to facilitate the prevention of buckling delamination. In addition, the delamination of the ITO-PET interface is highly possible when a PET with a thickness of 100 nm is introduced.

#### 4.3.3. Thickness Effect of ITO-Coating on PET Substrate

**Figure 14:**
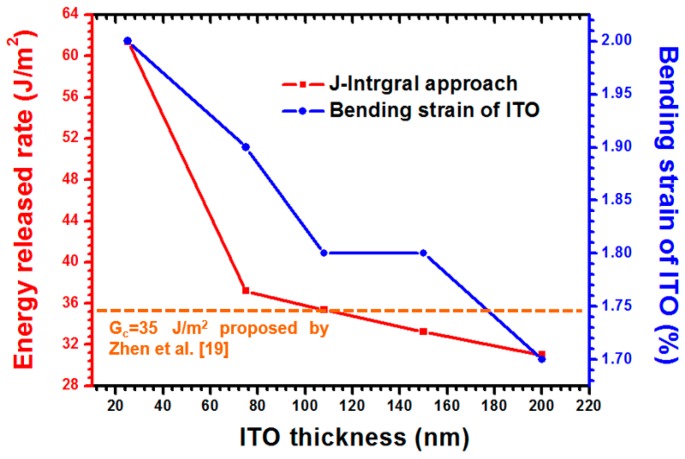
Effects of ITO thickness on energy release rate estimation regarding the interface of ITO-PET thin films and uniaxial strain occurring on the ITO surface.

As shown in Figure 14, the same simulation approach was also used to estimate the G_c_ and the bending strain of the ITO under various ITO thicknesses. For a small ITO with a 25-nm thickness, a 2.00% ITO bending strain was obtained because a reduced moment of inertia resulted from the decreased ITO thickness. In addition, the G-value of 61.4 J/m^2^ was estimated when the foregoing ITO thickness was achieved. This phenomenon can be attributed to the high bending strain resulting from a high strain energy release rate. Hence, a higher G-value was acquired to induce the buckling delamination when a thinner ITO film was analyzed. Therefore, the thickness of the ITO film substantially influenced the bending strain of the ITO and predicated the energy release rate of the ITO-PET interface.

## 5. Conclusions

In this study, compressive and tensile stress loads were exerted on an ITO-PET film by applying a bending cycling test to explore the influence of stress on the mechanical stability of the aforementioned stacked thin films. Flexible PET substrates with two different thicknesses were also prepared and experimented on to determine the thickness effect of the PET substrates. The sheet resistance of the ITO film increased whether the film was subjected to compressive or tensile stress, for which the tensile stress increased the sheet resistance of the ITO film more easily than the compressive stress did. Furthermore, this study revealed that an increase in the PET substrate thickness accelerated the failure occurrence of the ITO film. The sheet resistance of the ITO film notably increased when the film was deposited on a thicker PET substrate. In addition, the optical transmittance was extended under the statuses of both compressive and tensile stress when a thick PET substrate was applied. Furthermore, the experimental data regarding the interfacial energy release rate between the ITO-PET films were validated using the fracture-based FEA simulation. A highly flexible loading test induced the concerned fractured energy of the ITO film and the corresponding bending strain was observed when a thin ITO or PET was applied. In other words, a thick ITO or PET was preferable for avoiding the occurrence of buckling.

## Figures and Tables

**Table 1:** Properties of the materials used in the interfacial strength estimation of the ITO-PET film.

Materials	Young’s Modulus (GPa)	Poisson’s Ratio
ITO	118	0.3
PET	3.1	0.4
